# Embolization coils in treating postoperative bronchopleural fistula: a systematic review

**DOI:** 10.3389/fmed.2024.1364994

**Published:** 2024-06-20

**Authors:** Xiaojuan Luo, Ke Zhan, Yang Bai

**Affiliations:** ^1^Department of Endoscopy Center, The First Affiliated Hospital of Chongqing Medical University, Chongqing, China; ^2^Department of Gastroenterology, The First Affiliated Hospital of Chongqing Medical University, Chongqing, China; ^3^Department of Respiratory and Critical Care Medicine, The First Affiliated Hospital of Chongqing Medical University, Chongqing, China

**Keywords:** bronchopleural fistula, embolization coil, bronchoscopic treatment, systematic review, interventional pulmonology

## Abstract

**Objective:**

This study aims to comprehensively evaluate embolization coils in treating postoperative bronchopleural fistula (BPF).

**Methods:**

A systematic review based on PubMed, Embase, and The Cochrane Library studies was conducted. All cases receiving embolization coils in treating postoperative BPF were included. The primary outcome was the efficacy of embolization coils in achieving closure of postoperative BPF.

**Results:**

20 patients from 9 studies were included in this systematic review. A median number of 3 (range: 1–10) embolization coils with sealants obtained a complete closure rate of 80% in patients with postoperative BPF with sizes ranging from 2 to 3.1 mm. Three patients with BPF over 3 mm and one with multiple organ failure failed this treatment. Two cases of coil migration were reported without causing respiratory failure or fistula recurrence.

**Conclusion:**

Embolization coils might be considered a safe and effective bronchoscopic treatment for small postoperative BPF of less than 3 mm in size. More extensive and rigorous studies are needed to further evaluate and confirm the optimal use of embolization coils in the context of an alternative to surgical repair.

## Introduction

Postoperative bronchopleural fistula (BPF) presents a significant challenge for both patients and physicians, with a prevalence ranging from 2.1 to 9.2% and a mortality rate between 16.4 and 71.2% (1). Postoperative BPF-related complications, such as infected pleural fluid regurgitation, severe pneumonia, and respiratory failure, contribute to the high mortality rate even after the standard surgical repair (1). Treating postoperative BPF remains a compelling challenge for thoracic surgeons and pulmonologists. Bronchoscopic treatments have emerged as complementary or adjuvant modalities in managing postoperative BPF (2). Sealants, embolization coils, and ventricular septal defect occluders have shown promise in cases where patients are unable or unwilling to undergo surgical repair, depending on the size and location of the BPF (3–5). Different embolization coils, originally designed for vessel occlusion, have demonstrated excellent efficacy with few complications in managing postoperative BPF since the 1990s (6). Typically made of platinum or other materials, these coils have proven to be cost-effective, easy-to-perform, and minimally invasive bronchoscopic treatment options for small postoperative BPF (6–14). This study aims to evaluate the most recent clinical studies using embolization coils in treating postoperative BPF. These details can further contribute to our understanding of the efficacy and potential benefits of using embolization coils as a treatment option alternative to surgical repair for this challenging condition.

## Methods

### Literature search strategy

The search strategy to identify original studies on treating postoperative BPF with embolization coils was executed using the keywords “bronchopleural fistula” OR “air leak” OR “alveolar-pleural fistula” AND “coil” OR “coils.” The search was conducted from July 1990 to December 2023 using PubMed, Embase, and The Cochrane Library databases. Additionally, bibliographies were manually searched for relevant articles.

The inclusion criteria were studies reporting the management of postpneumonectomy or postlobectomy BPF with embolization coils. The exclusion criteria were studies not referencing embolization coils in treating postoperative BPF, reviews, conference abstracts, studies lacking necessary information, and studies written in languages other than English.

### Data extraction

Two independent reviewers (X.J.L. and K.Z.) utilized a standardized data abstraction form to collect detailed information on each patient’s demographics, medical history, postoperative BPF features (location and size), embolization coil details (the brand, size, and number of coils inserted, the method of coil insertion, the sealants after coil insertion), and clinical outcomes in treating postoperative BPF with embolization coils. Treatment success refers to the complete closure of the postoperative BPF using embolization coils without any air leak via chest tube drainage. Treatment failure indicated partial or no closure of the BPF despite the use of embolization coils, even with additional coils, sealants, or both. Any disagreements between the two reviewers were resolved by consensus discussions.

### Risk of bias in individual case reports

Some measures were implemented in this study to address potential biases inherent in individual case reports. The inclusion and exclusion criteria were clearly defined to reduce selection bias when including cases receiving embolization coils for the closure of postoperative BPF. The standardized data abstraction form was utilized by two independent reviewers to collect detailed information about each patient, minimizing bias and enhancing the reliability of data extraction. The disagreements in interpreting the data were resolved by consensus discussions, ensuring consistency and mitigating bias introduced by individual interpretations. The objective outcomes were evaluated by predetermined criteria, reducing subjective interpretation and potential bias in assessing the efficacy of the embolization coil.

## Results

### Study selection

**Figure 1:**
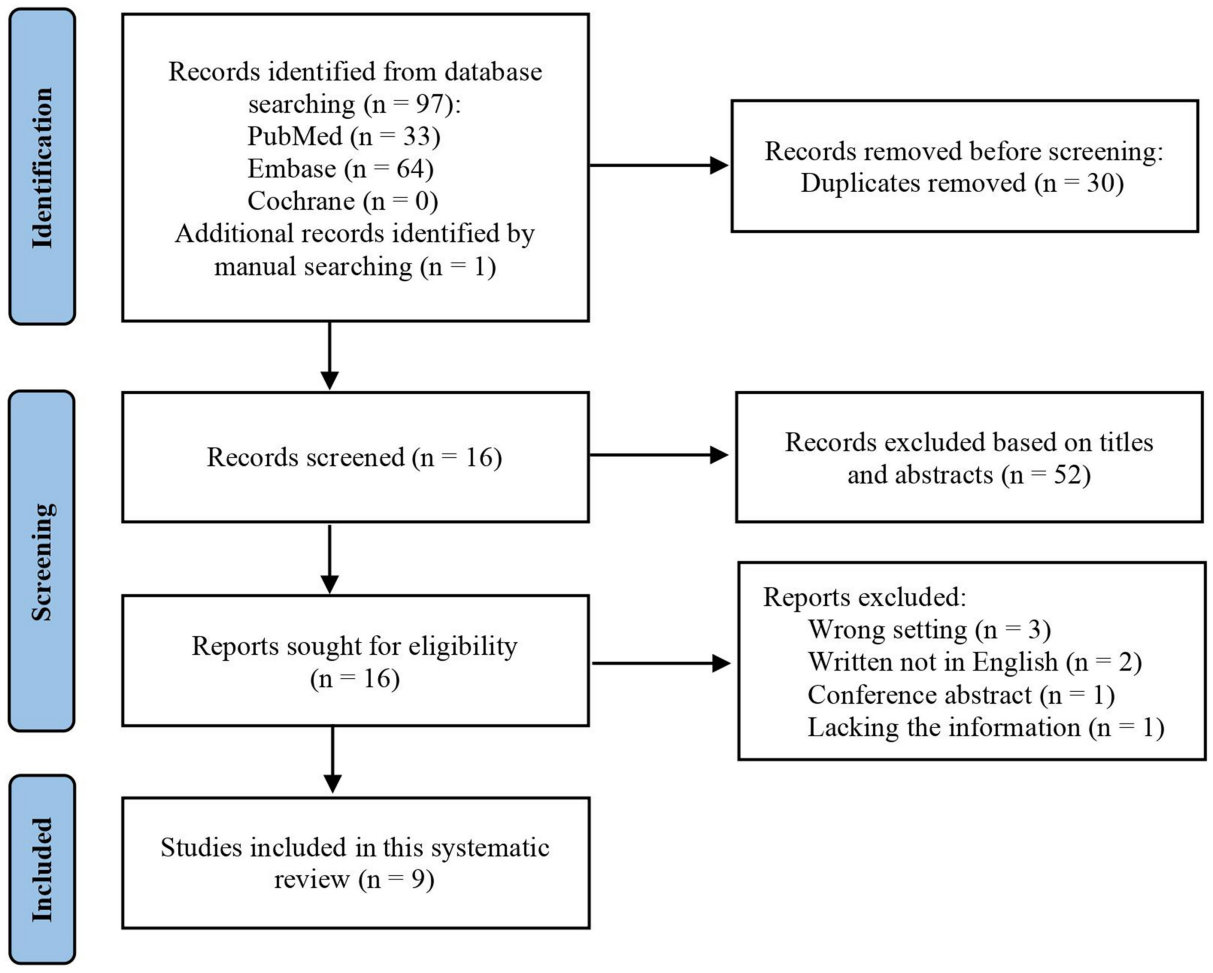
Literature flow diagram for embolization coils in treating postoperative bronchopleural fistula.

The literature search strategy identified 97 studies, which were disregarded for the following reasons: duplicated cases (*n* = 30), records excluded by titles and abstracts (*n* = 52) studies not referencing postoperative BPF (*n* = 3), conference abstracts (*n* = 1), studies lacking necessary information (*n* = 1), and studies written in languages other than English (*n* = 2). Nine studies reporting the management of postoperative BPF with embolization coils were included in this systematic review (Figure 1) (6–14). Supplementary Table S1 contains all the data extracted from these studies, providing further insight into treating postoperative BPF with embolization coils.

### Study characteristics

Table 1 summarizes the characteristics of the 20 patients for whom data were available from 9 studies. The median (range) age was 52 (33–80) years, and 64.3% of the population were male. The most prevalent cause for pneumonectomy or lobectomy was lung cancer (12/20, 60%), followed by tuberculosis-related lung diseases (3/20, 15%), other lung-related diseases (3/20, 15%) (such as sarcoidosis with massive hemoptysis, pulmonary sequestration, and breast cancer with lung metastasis), and cystic pulmonary hydatidosis (2/20, 10%). Postoperative BPF was found in the right upper lobe in 5 patients, the right lower lobe in 3 patients, the left upper lobe in 3 patients, the left lower lobe in 3 patients, the right main bronchus in 2 patients, the left main bronchus in 2 patients, and the bronchus intermedius in 2 patients, with a median size of 3.6 mm, ranging from 2 to 6 mm. In some cases, the initial attempts to close the postoperative BPF failed, including tube thoracostomy, surgical debridement, intercostal muscle flap, and other sealants such as fibrin glue and geofoam (Supplementary Table S1).

**Table 1:** Characteristics of postoperative BPF receiving embolization coils in included cases.

	n/N	Percentage
Demographics		
Age (years), median (range)	52 (33–80)^a^	
Male sex	9/14^b^	64.3%
Female sex	5/14^b^	35.7%
Underlying diseases		
Lung cancer	12/20	60%
Tuberculosis	3/20	15%
Cystic pulmonary hydatidosis	2/20	10%
Others	3/20	15%
BPF location		
Right upper lobe	5/20	25%
Right lower lobe	3/20	15%
Left upper lobe	3/20	15%
Left lower lobe	3/20	15%
Right main bronchus	2/20	10%
Left main bronchus	2/20	10%
Bronchus intermedius	2/20	10%
BPF size (mm), median (range)	3.1 (2–6)^c^	
Numbers of coils used (per patient), median (range)	3 (1–10)^d^	
Coil brands		
Gianturco Stainless Steel Coils	3/20	15%
Boston Scientific Platinum Coils	3/20	15%
COOK Spring Embolization Coils	13/20	65%
TRUFILL Pushable Coils	1/20	5%
Coil insertion methods		
Endoscopic and fluoroscopic guidance	3/20	15%
Endoscopic guidance	14/20	70%
CT guidance (percutaneously)	2/30	10%
Fluoroscopic guidance	1/20	5%
Sealants		
Fibrin glue	11/20	55%
NBCA + Lipiodol	4/20	20%
NBCA	3/20	15%
Fibrin glue, NBCA + Lipiodol	1/20	5%
Surgical cottons soaked in fibrin glue	1/20	5%
Follow-up months, median (range)	12 (1–24)^e^	
Outcomes		
Complete closure with coils initially	16/20	80%
Expectoration or dislodge of coils	2/20	10%
Recurrence of BPF	0/16	0%
Death not related to coil insertion	3/20	15%

a Ages of 5 patients were not reported.

b Genders of 6 patients were not reported.

c BPF sizes of 13 patients were not reported.

d Coil numbers of 10 patients were not reported.

e Follow-up time of 5 patients were not reported.

BPF: bronchopleural fistula; NBCA: N-butly-2-cyanoacrylate; CT: computer tomography.

The median number of embolization coils inserted per patient was 3 (range: 1–10). There were four different types of commercially available embolization coils utilized in these studies: COOK Spring Medical Coils in 13 patients, Gianturco Stainless Steel Coils in 3, Boston Scientific Platinum Coils in 3, and TRUFILL Pushable Coils in 1. In the majority of these patients (13/20, 65%), embolization coils were inserted under bronchoscopic observation, followed by endoscopic and fluoroscopic surveillance (3/20, 15%), computer tomography guidance percutaneously (2/20, 10%), and fluoroscopic inspection (1/20, 5%). After the insertion of embolization coils, different sealants were used in all patients, including fibrin glue, N-butly-2-cyanoacrylate, lipiodol, and surgical cotton, either alone or in combination.

### Efficacy and complications

The embolization coils with sealants achieved a complete closure rate of 80% (16/20) in patients with postoperative BPF, with sizes ranging from 2 to 3.1 mm (Table 1). In two cases, the subsequent insertion of longer coils attained complete closure following the initial attempt with short coils. Four cases with postoperative BPF had immediate closure after the insertion of embolization coils (Supplementary Table S1). Most of the studies did not report the closure time after coil insertion. The embolization coils failed in three patients with BPF sizes over 3 mm and one with multiple organ failure after the insertion of eight Gianturco Stainless Steel Coils into several distal bronchi (Supplementary Table S1, details). During a median follow-up period of 12 months (range, 1–24 months), there was no fistula recurrence in patients who achieved complete closure with the insertion of embolization coils. During the follow-up, one coil was expectorated out of the mouth without causing hypoxia, and another was dislodged into the thoracic cavity without causing fistula recurrence. Three patients passed away after the insertion of embolization coils, but not due to the procedure.

## Discussion

Postoperative BPF is a rare but severe consequence of pneumonectomy or lobectomy characterized by the formation of a pathological connection between the bronchial stump and pleural cavity, resulting in persistent air leakage, empyema, respiratory failure, and a high mortality rate (15). Surgical repair or additional resection should be recommended for BPF closure if patients are willing and able to endure the reoperation (16). However, surgical procedures for postoperative BPF closure were occasionally associated with a low success rate and a high overall mortality rate (17). Bronchoscopic intervention has evolved to diagnose and treat postoperative BPF with sealants, embolization coils, and ventricular septal defect occluders, depending on the size and location of the fistula (3–5). Embolization coils are initially used for permanent vessel occlusion without inducing ischemia in downstream vessels when deployed properly and precisely (18). They have been applied in limited cases for postoperative BPF closure since the 1990s (6). As far as we know, no previous systematic review has reported using embolization coils in treating postoperative BPF. This systematic review evaluated the most recent clinical studies on embolization coils in treating postoperative BPF. We observed that embolization coils with sealants achieved a complete closure in patients (16/20, 80%) with postoperative BPF ranging in size from 2 to 3.1 mm but failed in three patients with BPF sizes more than 3 mm and one with multiple organ failure. Embolization coils carry an overall success rate comparable to surgical procedures in treating postoperative BPF with a size less than 3 mm (19, 20).

The insertion of embolization coils in treating postoperative BPF is a safe procedure, according to the complications observed in the available literature. These complications included coil migration and expectoration without causing respiratory failure due to its modest size (7, 8). In this systematic review, no deaths were related to the insertion of embolization coils. The BPF size might be a major predictor of the prognosis and complications of bronchoscopic intervention with embolization coils in treating postoperative BPF (21, 22). Large postoperative BPF was associated with treatment failure with embolization coils and expectoration of fibrin glue (23). Unfortunately, many studies included did not report information on BPF size. The thin-section computer tomography could determine the size of postoperative BPF (the narrowest part of the fistula tract), which could help in evaluating treatment strategies and the diameter of the medical device used to close the fistula (24). The diameter of the initial embolization coil inserted should be 10–20% larger, or at least 2 mm oversized, than the fistula tract being occluded to increase the success rate and decrease the migration rate.

In the studies included in this systematic review, four different commercially available brands of pushable embolization coils were employed, with COOK Spring Medical Coils being the most prevalent. The pushable Nester^®^ Embolization Coils (Cook Medical) are made of platinum with spaced synthetic fibers, which can be delivered safely and effectively to the target location through the standard angiographic catheter with a flexible guide wire (25). Once delivered, it will form a tight occluding mass that anchors into the target location and occludes the vessel or fistula tract. Since the 1990s, embolization coils have been applied to treat postoperative BPF (6). The embolization coils generate a core for the occlusion of liquid sealants, thereby reducing the risk of cured sealant migration and fistula recurrence (6, 8, 12). As reported in porcine arteries, the synthetic fibers may cause local inflammation and promote the development of granulation tissue, thoroughly blocking postoperative BPF and probably reducing the number of coils required (26). The detachable coil was also used to successfully treat pneumonia-induced BPF in a two-year-old child (27). In managing postoperative BPF, the pushable Nester^®^ Embolization Coils are more cost-effective than the detachable coils because they are significantly less expensive (28).

The literature described four monitoring strategies for the insertion of embolization coils into a fistula: fluoroscopic inspection, endoscopic and fluoroscopic surveillance, computer tomography guidance percutaneously, and bronchoscopic observation (the most prevalent) (6–14). The precise occlusion of the fistula in the distal bronchial stump was made possible by the angiography catheter-guided or directed insertion of embolization coils under bronchoscopic observation and/or fluoroscopic inspection (6–10, 12–14). With the computer tomography-guided transthoracic needle, embolization coils could be inserted transversally into the fistula tract, and cyanoacrylate glue could be injected into and adjacent to the fistula (11). Although there is no risk of pneumothorax with the percutaneous insertion of embolization coils under computer tomography guidance in patients with postoperative BPF, there is still a risk of hemorrhage. We prefer not to insert the embolization coils percutaneously under computer tomography guidance for postoperative BPF in patients tolerating bronchoscopy.

Several limitations in this systematic review must be acknowledged. The interpretation of the present findings was predominantly constrained by the small sample size and the heterogeneity of the case reports, which makes it difficult to reach a definitive conclusion. Studies demonstrating positive outcomes are more likely to be published, which could lead to an overestimation of the efficacy of embolization coils in treating postoperative BPF. The evidence-based data on how to treat postoperative BPF is still limited, consisting mainly of case reports and case series. Well-designed prospective studies, randomized controlled trials, or cohort studies with larger sample sizes and standardized methodologies are needed to evaluate the efficacy of embolization coils in treating postoperative BPF, especially with long-term follow-up data.

## Conclusion

In conclusion, with a high success rate and low complication rate, embolization coils might offer a minimally invasive, cost-effective, and relatively easy-to-perform alternative to surgical repair for small postoperative BPF (less than 3 mm in size). Not all patients with postoperative BPF are appropriate candidates for coil embolization. The decision to apply this technique should be made on a case-by-case basis, considering factors such as the size and location of the fistula and the patient’s overall health status. Further studies are needed to evaluate their long-term efficacy and safety.

## Data availability statement

The original contributions presented in the study are included in the article/Supplementary material, further inquiries can be directed to the corresponding author.

## Author contributions

XL: Data curation, Investigation, Writing – original draft. KZ: Data curation, Investigation, Writing – original draft. YB: Conceptualization, Investigation, Writing – original draft, Writing – review & editing.
