# Supplementary material for: Embolization coils in treating postoperative bronchopleural fistula: a systematic review
Source: Front Med (Lausanne). 2024 Jun 20;11:1364994. doi: 10.3389/fmed.2024.1364994 (PMC11222595; doi:10.3389/fmed.2024.1364994)
Supplement: Supplementary file 1 [file Table_1.pdf]

Supplementary Table 1. Details of postoperative BPF treated with embolization coils in included cases

Reference	Age (yrs) / sex (M/F)	Medical history	Previous treatments	BPF locations	BPF size (mm)	Pushable coil brands	Coil details	Coil insertion methods	Sealants	Outcomes	Closure time after coil insertion (days)	Follow-up time (months)	Complications and follow-up
Salmon et al 1990 [1]	41/M	Sarcoidosis and massive hemoptysis, right upper and middle lobectomies	Fibrin glue and gelfoam	Superior segments of the RLL	NR	Gianturco Steel Coils	5mm×3	Endoscopic and fluoroscopic guidance	Fibrin glue	Complete closure	10	18	No recurrence of hemoptysis or fistula
Ponn et al 1993 [2]	78/M	LUAD, left upper lobectomy	Tube thoracostomy	Superior segments of the LLL	NR	Gianturco Steel Coils	5mm×2	Endoscopic and fluoroscopic guidance	Fibrin glue when necessary	Complete closure	Immediately after coil insertion	12	One coil was expectorated 4 months later without causing symptoms
	71/M	Synchronous adenocarcinoma, transsternal right lower lobectomy and wedge of excision of the left upper lobe, multiple organ failure	Tube thoracostomy	Posterior segment of the RUL	NR	Gianturco Steel Coils	8mm×3, 5mm×2, 3mm×3	Endoscopic and fluoroscopic guidance	Fibrin glue when necessary	Failure	-	2.5	The patient died of multiple organ failure
Hirata et al 2002 [3]	73/M	LUSC, right middle and lower lobectomies, right pneumonia and pyothorax	Tube thoracostomy, surgical debridement and intercostal muscle flap, and fibrin glue	Anterior segment of the RUL	NR	Boston Scientific Platinum Coils	6mm×3+ 5mm×1 (first), and 30mm×1+50mm×1 (second)	Endoscopic guidance	Fibrin glue, NBCA+Lipiodol	Complete closure (after subsequent insertion of long coils) [#]	Immediately after coil insertion	10	The patient died 10 months for respiratory failure due to another newly developed fistula.
	64/F	Left breast cancer, left pulmonary metastasis, partial resection of the left lung and right subsegmentectomy	Tube thoracostomy, pleurodesis, and intercostal muscle flap	Posterior segment of the RUL	NR	Boston Scientific Platinum Coils	4mm×7 (first), 10mm×2+4mm×1 (second)	Endoscopic guidance	NBCA	Complete closure (after subsequent insertion of long coils)	Immediately after coil insertion	6	No recurrence of fistula
Watanabe et al. 2003 [4]	NR/NR	Lung cancer, right middle and lower lobectomy	NR	Bronchus intermedius	NR	COOK Spring Medical Coils	NR	Endoscopic guidance	Fibrin glue	Complete closure	NR	NR	No recurrence of fistula
	NR/NR	Lung cancer, right lower lobectomy	NR	Segmental bronchi of the RUL	NR	COOK Spring Medical Coils	NR	Endoscopic guidance	Fibrin glue	Complete closure	NR	NR	No recurrence of fistula
	NR/NR	Lung cancer, left lower lobectomy	NR	Segmental bronchi of the LUL	NR	COOK Spring Medical Coils	NR	Endoscopic guidance	NBCA+Lipiodol	Complete closure	NR	NR	No recurrence of fistula
	NR/NR	Lung cancer, left upper lobectomy	NR	Segmental bronchi of the LUL	NR	COOK Spring Medical Coils	NR	Endoscopic guidance	NBCA+Lipiodol	Complete closure	NR	NR	No recurrence of fistula
	NR/NR	Lung cancer, left pneumonectomy	NR	LMB	> 6	COOK Spring Medical Coils	NR	Endoscopic guidance	NBCA+Lipiodol	Failure	-	NR	No recurrence of fistula
Shimizu et al. 2005 [5]	80/M	LUSC, right middle and lower lobectomy	Tube thoracostomy	Bronchus intermedius	NR	TRUFILL Pushable Coils	7cm×4	Endoscopic guidance	Surgical cottons soaked in fibrin glue	Complete closure	Immediately after coil insertion	14	No recurrence of fistula
Clemson et al. 2006 [6]	57/M	LUAD, right pneumonectomy, pathologic fracture of the right pelvis from metastasis	NR	RMB	NR	COOK Spring Medical Coils	8mm×2+8mm×2	CT guidance, coil inserted percutaneously)	NBCA	Complete closure (after the second insertion of coils)	NR	1	The patient died due to systematic metastasis
	47/F	NSCLC, right pneumonectomy, four cycles of chemotherapy	NR	RMB	NR	COOK Spring Medical Coils	8mm×1	CT guidance, coils inserted percutaneously	NBCA	Complete closure	5	1	No recurrence of fistula
Sivrikoz et al. 2007 [7]	59/M	LUAD, left pneumonectomy	Tube thoracostomy	LMB	3	Boston Scientific Platinum Coils	5mm×3	Endoscopic guidance	Fibrin glue	Complete closure	NR	12	No recurrence of fistula
Bae et al. 2010 [8]	40/NR	Pulmonary sequestration, left lower lobectomy	Tube thoracostomy	Inferior lingular segment of the LUL	NR	COOK Spring Medical Coils	3mm-2cm×2	Fluoroscopic guidance	NBCA+Lipiodol	Complete closure	3	12	No recurrence of fistula
Marwah et al. 2020 [9]	42/F	Post tubercular bronchiectasis with hemoptysis, right lower lobectomy	NR	RLL	3.8	COOK Spring Medical Coils	NR	Endoscopic guidance	Fibrin glue	Failure	-	24	Recurrence of fistula, and later stump closed surgically
	33/M	Cystic pulmonary hydatidosis, left lower lobectomy	NR	LLL	2	COOK Spring Medical Coils	NR	Endoscopic guidance	Fibrin glue	Complete closure	NR	12	No recurrence of fistula
	38/M	Cystic pulmonary hydatidosis, left lower lobectomy	NR	LLL	3.1	COOK Spring Medical Coils	NR	Endoscopic guidance	Fibrin glue	Complete closure	NR	15	No recurrence of fistula
	52/F	Post tubercular sequelae with Aspergilloma and hemoptysis, right upper lobectomy	NR	RUL	3.6	COOK Spring Medical Coils	NR	Endoscopic guidance	Fibrin glue	Failure	-	24	Recurrence of fistula, and later stump closed surgically
	48/F	Tuberculous empyema (right) not responding to conservative management, right lower lobectomy	NR	LLL	2	COOK Spring Medical Coils	NR	Endoscopic guidance	Fibrin glue	Complete closure	NR	24	No recurrence of fistula

One short coil fell into thoracic cavity with fistula recurrence, and two long coils closed it.
BPF: bronchopleural fistula; LLL: left lower lobe; LMB: left main bronchus; LUL: left upper lobe; LUAD: lung adenocarcinoma; LUSC: lung squamous cell carcinoma; RLL: right lower lobe; RMB: right main bronchus; RUL: right upper lobe; NR: not reported; NBCA: N-butly-2-cyanoacrylate.

Reference

1. Salmon CJ, Ponn RB, Westcott JL. Endobronchial vascular occlusion coils for control of a large parenchymal bronchopleural fistula. *Chest.* 1990;98(1):233-234.
2. Ponn RB, D'Agostino RS, Stern H, Westcott JL. Treatment of peripheral bronchopleural fistulas with endobronchial occlusion coils. *The Annals of thoracic surgery.* 1993;56(6):1343-1347.
3. Hirata T, Ogawa E, Takenaka K, Uwokawa R, Fujisawa I. Endobronchial closure of postoperative bronchopleural fistula using vascular COOKcoils and n-butyl-2-cyanoacrylate. *The Annals of thoracic surgery.* 2002;74(6):2174-2176.
4. Watanabe S, Watanabe T, Urayama H. Endobronchial occlusion method of bronchopleural fistula with metallic coils and glue. *The Thoracic and cardiovascular surgeon.* 2003;51(2):106-108.
5. Shimizu J, Takizawa M, Yachi T, et al. Postoperative bronchial stump fistula responding well to occlusion with metallic coils and fibrin glue via a tracheostomy: a case report. *Annals of thoracic and cardiovascular surgery : official journal of the Association of Thoracic and Cardiovascular Surgeons of Asia.* 2005;11(2):104-108.
6. Clemson LA, Walser E, Gill A, Lynch JE, Zwischenberger JB. Transthoracic closure of a postpneumonectomy bronchopleural fistula with coils and cyanoacrylate. *The Annals of thoracic surgery.* 2006;82(5):1924-1926.
7. Sivrikoz CM, Kaya T, Tulay CM, Ak I, Bilir A, Döner E. Effective approach for the treatment of bronchopleural fistula: application of endovascular metallic ring-shaped coil in combination with fibrin glue. *The Annals of thoracic surgery.* 2007;83(6):2199-2201.
8. Park JD, Bae IH, Park KS, Kim SJ, Jeon MH, Hong JM. Fluoroscopy-Guided Treatment of a Bronchopleural Fistula with a Platinum Vascular Occlusion Coil and N-butly-2-cyanoacrylate (NBCA): A Case Report. *jksr.* 2009;61(6):375-378.
9. Marwah V, Katoch CDS, Kumar K, Pathak K, Bhattacharjee S, Jindamwar P. Bronchoscopic device closure of postoperative bronchopleural fistulae: Novel devices and innovative techniques. *Lung India : official organ of Indian Chest Society.* 2020;37(2):107-113.
